# Corticotrigeminal Projections from the Insular Cortex to the Trigeminal Caudal Subnucleus Regulate Orofacial Pain after Nerve Injury via Extracellular Signal-Regulated Kinase Activation in Insular Cortex Neurons

**DOI:** 10.3389/fncel.2015.00493

**Published:** 2015-12-24

**Authors:** Jian Wang, Zhi-Hua Li, Ban Feng, Ting Zhang, Han Zhang, Hui Li, Tao Chen, Jing Cui, Wei-Dong Zang, Yun-Qing Li

**Affiliations:** ^1^Department of Anatomy and K. K. Leung Brain Research Centre, Fourth Military Medical UniversityXi’an, China; ^2^Basic Medical College, Zhengzhou UniversityZhengzhou, China; ^3^Collaborative Innovation Center for Brain Science, Fudan UniversityShanghai, China

**Keywords:** insular cortex (IC), trigeminal caudal subnucleus (Vc), extracellular signal-regulated kinase (ERK), IoN-CCI, neuropathic pain

## Abstract

Cortical neuroplasticity alterations are implicated in the pathophysiology of chronic orofacial pain. However, the relationship between critical cortex excitability and orofacial pain maintenance has not been fully elucidated. We recently demonstrated a top-down corticospinal descending pain modulation pathway from the anterior cingulate cortex (ACC) to the spinal dorsal horn that could directly regulate nociceptive transmission. Thus, we aimed to investigate possible corticotrigeminal connections that directly influence orofacial nociception in rats. Infraorbital nerve chronic constriction injury (IoN-CCI) induced significant orofacial nociceptive behaviors as well as pain-related negative emotions such as anxiety/depression in rats. By combining retrograde and anterograde tract tracing, we found powerful evidence that the trigeminal caudal subnucleus (Vc), especially the superficial laminae (I/II), received direct descending projections from granular and dysgranular parts of the insular cortex (IC). Extracellular signal-regulated kinase (ERK), an important signaling molecule involved in neuroplasticity, was significantly activated in the IC following IoN-CCI. Moreover, in IC slices from IoN-CCI rats, U0126, an inhibitor of ERK activation, decreased both the amplitude and the frequency of spontaneous excitatory postsynaptic currents (sEPSCs) and reduced the paired-pulse ratio (PPR) of Vc-projecting neurons. Additionally, U0126 also reduced the number of action potentials in the Vc-projecting neurons. Finally, intra-IC infusion of U0126 obviously decreased Fos expression in the Vc, accompanied by the alleviation of both nociceptive behavior and negative emotions. Thus, the corticotrigeminal descending pathway from the IC to the Vc could directly regulate orofacial pain, and ERK deactivation in the IC could effectively alleviate neuropathic pain as well as pain-related negative emotions in IoN-CCI rats, probably through this top–down pathway. These findings may help researchers and clinicians to better understand the underlying modulation mechanisms of orofacial neuropathic pain and indicate a novel mechanism of ERK inhibitor-induced analgesia.

## Introduction

The orofacial region is often affected by chronic neuropathic pain conditions. Following orofacial surgical treatment such as third molar extraction, neuropathic pain, in addition to trigeminal neuralgia, sometimes occurs in the orofacial region and is difficult to diagnose and treat (Truelove, 2004; Zakharov et al., 2015). Additionally, chronic orofacial pain can induce anxiety/depression-like negative emotions, which in turn further exaggerate patients’ pain sensations (Logan et al., 2003; Eboli et al., 2009). Although significant progress has been made, the currently available therapies for orofacial neuropathic pain remain inadequate, and the underlying mechanisms have not been completely elucidated.

Orofacial nociceptive information is preliminarily transmitted to the trigeminal caudal subnucleus (Vc) in the brainstem and then ascends to higher brain regions (Dubner and Bennett, 1983). Similar to that in the spinal cord, orofacial nociceptive transmission in the Vc of the brainstem also receives descending inhibitory and facilitatory modulations from supraspinal structures such as the periaqueductal grey (PAG) and rostral ventromedial medulla (RVM) (Dubner and Bennett, 1983; Okubo et al., 2013; Wang et al., 2014; Feng et al., 2015). Our group previously reported a direct top-down corticospinal pathway in which neurons in the anterior cingulate cortex (ACC) sent descending projections to the spinal cord and regulated pain transmission (Chen et al., 2014a,b). Thus, in addition to PAG-RVM-Vc descending modulation pathways, a top-down pathway that originates from higher brain regions such as the cortex may directly regulate neurons of the Vc.

Recently, the insular cortex (IC) has been increasingly reported to play important roles in several major brain functions, including awareness, fear memory, and pain (Craig et al., 2000; Henderson et al., 2007; Zhuo, 2008). According to *in vivo* or *in vitro* electrophysiological recordings, IC neurons are activated under acute and chronic pain conditions (Liu et al., 2013; Qiu et al., 2013). Brain lesion studies show that inhibiting IC activity produces analgesia (Wei et al., 2001; Coffeen et al., 2011). Moreover, human brain imaging data suggest that the IC is activated by noxious stimuli, and direct electrical stimulation of the IC elicits painful sensations; these findings support the critical roles of the IC in pain perception (Mazzola et al., 2009, 2012). Long-term potentiation (LTP) in synaptic transmission is widely accepted as the key cellular mechanism for not only learning and memory but also storing pain information in the brain (Kandel, 2001; Li et al., 2010). LTP of the IC is protein synthesis dependent and requires the activation of N-methyl-d-aspartate (NMDA) receptors (Kandel, 2001). Furthermore, activity-dependent plasticity occurs in the IC under nerve injury-induced neuropathic pain conditions (Qiu et al., 2013).

Extracellular signal-regulated kinase (ERK) belongs to the mitogen-activated protein kinase (MAPK) family and transduces extracellular stimuli into intracellular responses in a wide variety of circumstances (Widmann et al., 1999). Neural ERK plays important roles in synaptic plasticity and remodeling during LTP, learning, and memory consolidation (Sweatt, 2001). According to previous studies, inflammation or nerve injury activates the ERK pathway not only in the spinal dorsal horn but also in supraspinal structures such as the medial prefrontal cortex (mPFC), ACC, and IC (Ji et al., 1999; Wei and Zhuo, 2008; Alvarez et al., 2009; Xu et al., 2014). Thus, ERK activity is important for synaptic plasticity in the brain during the induction and expression of various types of pain.

Given the importance of ERK activity in the synaptic plasticity under pain conditions and the possible existence of a direct corticotrigeminal descending modulation pathway, in the current study, we hypothesized that ERK activity in the IC contributed to infraorbital nerve chronic constriction injury (IoN-CCI)-induced orofacial pain in rats. In the present study, we demonstrated that IoN-CCI-induced neuropathic pain led to the long-term activation of phosphorylated ERK (p-ERK) in the IC. Moreover, the IC could send direct projections primarily to the contralateral Vc. More importantly, ERK inhibition via U0126 perfusion of IC slices *in vitro* not only inhibited the spontaneous activity of Vc-projecting neurons in the IC but also reduced their excitability. Finally, intra-IC microinjection of U0126 significantly decreased Fos expression in the Vc and effectively alleviated established nociceptive behaviors as well as pain-related negative emotions in IoN-CCI rats.

## Materials and Methods

### Animals

Adult male Sprague-Dawley (SD) rats (weighing 250–300 g) were housed in standard plastic cages with a 12: 12-h light/dark cycle (light on at 08:00 a.m.) under 22–25°C ambient temperature and were provided food and water *ad libitum*. The Ethics Committee for Animal Experiments of the Fourth Military Medical University (Xi’an, China) approved the animal experiments (Permit number: 10071). All procedures were in agreement with the IASP guidelines (Zimmermann, 1983). Efforts were made to minimize the number of animals used and their suffering.

### Establishment of IoN–CCI Model

IoN-CCI was performed following a previously established surgical procedure (Wei et al., 2008). Briefly, rats were anesthetized with pentobarbital sodium (45 mg/kg, i.p.), and loose ligatures of the right IoN were performed via an intraoral approach. An 8-10-mm-long incision was made along the gingivobuccal margin in the buccal mucosa, beginning immediately next to the first molar. The IoN was loosely tied with two chromic gut (5-0) ligatures, 2 mm apart, 3–4 mm from the nerve, where it emerges from the infraorbital foramen, to induce minor constriction of the IoN; the superficial vasculature was minimally disturbed. The incision was closed using 4-0 silk sutures. The surgical procedures for the sham-operated group were identical to those for the IoN-CCI group, except that the nerves were not ligatured. In all cases, an anesthetic agent of 0.5% lidocaine was injected at the incision sites three times per day for 2 days after CCI or sham surgery to block local nociceptive inputs induced by acute tissue injury.

### Behavioral Tests

All the behavioral tests were conducted in a quiet room under dim light (30 lux) during the early light phase (9 a.m.–1 a.m.) of the light cycle by an observer who was blinded to the treatment groups.

#### Nociceptive Behavioral Tests

Mechanical allodynia, as a behavioral sign of IoN-CCI-induced neuropathic pain, was assessed by measuring the 50% head withdrawal threshold (HWT), as previously described (Geng et al., 2010). Rats were habituated to the testing for 3 days before baseline testing and were then placed in a small handmade porous metal mesh cage to habituate for 30 min prior to the threshold test. The 50% HWT in response to von Frey filaments (Stoelting, Kiel, WI, USA) was determined by a previously reported method (Okubo et al., 2013). Briefly, when the rat’s head was steadily resting or in alert status, a series of ascending von Frey filaments were inserted through the mesh wells from the lateral side. The von Frey filaments were delivered to the skin near the center of the vibrissa pad within the infraorbital territory for 2–3 s. Brisk or active withdrawal of the head from the probing filament was defined as a positive response.

#### Open Field Test

The open field (OF) test was conducted as described in previous studies (Sun et al., 2013; Wu et al., 2014). Rats were placed at the center of a cubic chamber (100 cm × 100 cm × 60 cm). The locomotion of rats in 10 min was monitored by an automated analyzing system (Shanghai Mobile Datum Information Technology, Shanghai, China). The total distance traveled was used as a parameter for evaluating locomotion, and the percentage of time spent in the center area (center time%) was used as a parameter for evaluating anxiety/depression levels (Prut and Belzung, 2003; Campos et al., 2013) by off-line analysis. All animals were habituated to the testing room for 30 min before the OF test.

#### Elevated Plus Maze Test

The elevated plus maze (EPM) comprised two open arms (OAs; 50 cm × 10 cm) and two enclosed arms (CAs; 50 cm × 10 cm × 40 cm) that extended from a common central platform (10 cm × 10 cm). The plus-shaped platform was 50 cm above the floor. Generally, rats were placed individually into the center area of the maze facing one of the OAs and were allowed to explore for 5 min. The numbers of OA and CA entries and the times spent in the OAs and CAs were recorded by an automated analyzing system (Shanghai Mobile Datum Information Technology). The animal was considered to be in an OA or CA only when all four paws crossed out of the central zone (Zhai et al., 2015). The EPM test relies on the animal’s natural fear of open spaces, and the percent of time spent in OAs (OA time%) and the percent of OA entries (OA entries%) are considered measurements of general anxiety/depression levels (Rainer et al., 2014). OA time% = OA time/(OA time + CA time). OA entries% = OA entries/(OA entries + CA entries) (Rainer et al., 2014; Wu et al., 2014).

### Retrograde and Anterograde Tract Tracing

To investigate the fiber connections between the Vc and the IC in rats, we performed retrograde and anterograde tract tracing experiments. Fluoro-Gold (FG) was used as a retrograde tracer to label the IC neurons that project to the Vc. In addition, in the electrophysiological study, tetramethylrhodamine-dextran (TMR) was used to label Vc-projecting neurons in the IC. The procedures for FG/TMR injection were identical to those of our previous study (Wang et al., 2014). Briefly, after the cisternal cavity of the caudal medulla oblongata was exposed, 0.1 μl of a 4% (w/v) solution of FG (dissolved in 0.9% saline; Fluorochrome, Denver, CO, USA) or a 10% (w/v) solution of TMR (dissolved in 0.1 M citrate-NaOH, pH 3.0; Molecular Probes, Eugene, OR, USA) was stereotaxically pressure-injected into the right Vc through a glass micropipette, which was attached to a 1-μl Hamilton microsyringe. The rats were allowed to recover for 6 days before they were perfused.

For the anterograde tract tracing, the anterograde tracer Phaseolus vulgaris-leukoagglutinin (PHA-L) was used to identify the Vc-projecting fibers and the terminals originating from the IC. The procedures for PHA-L injection were essentially the same as previously described by our group (Li et al., 1993; Chen et al., 2014a). Briefly, PHA-L (Vector Laboratories, Burlingame, CA, USA) was dissolved in a mixture of 0.05 M Tris-HCl buffer and 0.5 M KCl (pH 7.6) to a final concentration of 2.5% (w/v). PHA-L was iontophoretically (positive, 3–5 μA, 7 s on/off, 25 min) injected into unilateral deep layers of the IC according to the rat brain atlas (0.36 mm posterior to bregma, 6.2 mm lateral to the midline and 4.1 mm deep from the cerebral surface). After injection, the surgical wounds were carefully sutured. Rats were allowed to survive for approximately 2 weeks before perfusion.

### Cannula Implantation and Microinjection into the IC

After the rats were anesthetized with pentobarbital (45 mg/kg, i.p.), a 26-gage stainless steel guide cannula was stereotaxically implanted into a site above the bilateral IC. The rats were allowed to recover for 1 week following cannula implantation. Intra-IC microinjections were delivered via a 33-gage injector needle cannula that was lowered 0.5 mm deeper into the brain than the guide cannula. The microinjection apparatus comprised a Hamilton syringe (10 μl) connected to an injector (33-gage) by a thin polyethylene tube and a motorized syringe pump. 1,4-Diamino-2,3-dicyano-1,4-bis (2-aminophenylthio) butadiene (U0126; Tocris Bioscience, Minneapolis, MN, USA) was selected to inhibit ERK activation, based on previous reports (Carrasquillo and Gereau, 2007). U0126 was first dissolved in 100% dimethylsulfoxide (DMSO) to a final stock concentration of 10 mM. On the day of the experiment, U0126 was diluted 1:1 in 0.9% saline to a final concentration of 5 mM in 50% DMSO/saline. For all experiments, 50% DMSO/saline was used as a vehicle control. A total volume of 0.5 μl drug or vehicle was infused into the IC at a rate of 0.1 μl/min. After injection, the microinjection needle was left in place for at least 2 min. The injection sites were verified at the end of all of the experiments by Nissl staining; injection sites outside the IC region were excluded from the study. A total of 32 rats showed successful implantation in the target.

To demonstrate whether U0126 could prevent IoN-CCI-induced neuropathic pain, the effects of U0126 on mechanical thresholds were measured before IoN-CCI (Pre-CCI), 13 days after IoN-CCI (CCI-D13), and 1 h after intra-IC drug or vehicle infusion 14 days after IoN-CCI (CCI-D14) (**Figure 6A**). We also investigated the effects of U0126 on the OF and EPM tests to assess changes in anxiety/depression-like behaviors 1 h after intra-IC U0126 injection on CCI-D14.

### Immunohistochemical Staining and Observation

The rats were transcardially perfused with 150 ml of 0.01 M phosphate-buffered saline (PBS, pH 7.4), followed by 500 ml of 4% paraformaldehyde in 0.1 M phosphate buffer (PB, pH 7.4). The brains and/or brainstems were transversely sliced into 25-mm-thick coronal sections using a freezing microtome (CM1950, Leica, Heidelberg, Germany).

Sections of the FG injection group were observed for the FG injection site in the Vc and for the distribution of retrogradely labeled neurons in the IC by using an epifluorescence microscope (BX-60; Olympus, Tokyo, Japan).

Immunohistochemical staining for FG, PHA-L, p-ERK, and Fos was performed with standard protocols. Briefly, after incubation with 3% H_2_O_2_ for 10 min, sections were washed with 0.01 M PBS three times for 10 min each and then incubated with 10% normal donkey serum (NDS) for 30 min. For different purposes, sections containing the IC or Vc regions were sequentially incubated with the following: primary antibodies in 0.01 M PBS containing 5% NDS, 0.3% Triton X-100, 0.05% NaN_3_, and 0.25% carrageenan (PBS-NDS, pH 7.4) for 24 h; biotinylated secondary antibodies in PBS-NDS at room temperature for 6 h; and avidin-biotin-peroxidase complex (ABC) (ABC Elite Kit; 1:200; Vector Laboratories) or fluorophore-conjugated avidin D in PBS at room temperature for another 2 h.

The following antibodies were used in the current study: guinea pig anti-FG (1:200; Protos Biotech, New York, NY, USA); rabbit anti-PHA-L (1:500; E-Y Laboratories, San Mateo, CA, USA); rabbit anti-p-ERK (1:200; Cell Signaling Technology, Danvers, MA, USA); mouse anti-Fos (1:500; Abcam, Cambridge, MA, USA); biotinylated donkey anti-guinea pig IgG (1:500, Vector Laboratories, Burlingame, CA, USA); biotinylated donkey anti-rabbit IgG (1:500; Vector Laboratories); biotinylated donkey anti-mouse IgG (1:500; Vector Laboratories); ABC Elite Kit (1:200; Vector Laboratories); and FITC-avidin D (1:1000; Vector Laboratories).

The PHA-L injection sites and the distributions of anterogradely PHA-L-labeled fibers and terminals, FG-retrogradely labeled neurons, and p-ERK-immunoreactive (IR) neurons were processed by using the ABC method. They were then visualized with diaminobenzidine (DAB) chromogen. Sections were then observed under a light microscope (AH-3; Olympus, Tokyo, Japan).

For the immunofluorescent staining of Fos, the sections were observed, and images were captured using a confocal laser-scanning microscope (CLSM, FV1000, Olympus). Digital images were captured using FLUOVIEW software (Olympus).

The negative control experiment, performed by replacing the primary antibody with 1% NDS in the protocol, exhibited no staining.

We randomly selected five 25-μm-thick sections from each rat (*n* = 4 rats; total 20 sections) and then counted the number of cells within the targeted brain regions. The p-ERK-IR neurons in the IC and Fos-IR neurons in the Vc were manually counted by an observer who was blinded to the treatment conditions, and the numbers of counted cells were corrected by using Abercrombie’s equation: number of cells = number of cells counted × T/(T + h), where T = thickness of the sections and h = the mean diameter of the nuclei of the large or small cells (Guillery, 2002).

### Western Blot Assay

According to the standard Western blot protocol, rats were anesthetized with an overdose of pentobarbital (60 mg/kg, i.p.), and the IC regions were carefully dissected and harvested for Western blotting. To obtain total protein extracts, the tissues were lysed in 300 μl lysis buffer containing 10 mM Tris, 150 mM NaCl, 1% Triton X-100, 0.5% NP-40, and 1 mM EDTA at pH 7.4. The samples were adequately mixed at a with protease inhibitor cocktail and phosphatase inhibitor cocktail (Roche, Tucson, AZ, USA) at a 100:1 (v/v) ratio. Subsequently, 30 μg of cell lysis material (quantitatively measured using the BCA protein assay; Thermo Scientific; Rockford, IL, USA) was resolved by sodium dodecyl sulfate-polyacrylamide gel electrophoresis (SDS-PAGE) and transferred to polyvinylidene fluoride (PVDF) membranes (Immobilon-P, Millipore, Temecula, CA, USA). After blocking in non-fat milk for 1 h, the membranes were incubated overnight at 4°C with the following primary antibodies: rabbit anti-ERK (1:1000, Cell Signaling Technology); rabbit anti-p-ERK (1:1000, Cell Signaling Technology); rabbit anti-CREB (1:1000, Cell Signaling Technology); rabbit anti-p-CREB (1:1000, Cell Signaling Technology); and mouse anti-β-actin (1:5000, Sigma, St. Louis, MO, USA). The immunoblots were then reacted with the corresponding horseradish peroxidase (HRP)-conjugated secondary antibodies (goat anti-rabbit 1:5000 goat anti-mouse 1:5000; Amersham Pharmacia Biotech, Piscataway, NJ, USA). All of the reactions were detected by the enhanced chemiluminescence (ECL) detection method (Amersham Pharmacia Biotech) and exposure to film. The scanned images were quantified and analyzed with ImageJ software. Target protein levels were normalized against β-actin levels and expressed as fold changes relative to those of the naive control group.

### Electrophysiology

Ten days after the IoN-CCI surgery, the rats were injected with TMR into the Vc ipsilateral to the side of nerve injury and allowed to recover for 4 days before the electrophysiological experiments were performed. The rats were decapitated, and coronal slices (400 mm) containing the contralateral IC were cut at 0°C on a vibrating microtome (VT1200s, Leica) in a sucrose cutting solution that was bubbled with carbogen gas (95% O_2_/5% CO_2_) and contained the following (in mM): 2.5 KCl, 1.2 NaH_2_PO_4_, 26 NaHCO_3_, 252 sucrose, 6 MgSO_4_, 0.5 CaCl_2_, and 10 glucose, pH 7.4). Then, the brain slices were transferred to a submerged recovery chamber with oxygenated artificial cerebrospinal fluid (ACSF) containing the following (in mM): 124 NaCl, 2.5 KCl, 2 MgSO_4_, 1 NaH_2_PO_4_, 25 NaHCO_3_, 2 CaCl_2_, and 37 glucose. The slices were incubated for 2 h at room temperature before recording.

The slices were transferred to a recording chamber (volume: 0.5 ml) that was mounted on a fixed-stage upright microscope (BX51W1, Olympus). Whole-cell patch-clamp recordings were made from TMR-containing Vc-projecting neurons that were visualized under epifluorescence using a tetramethyl rhodamine isothiocyanate (TRITC) filter set (U-HGLGPS, Olympus) with a monochrome CCD camera (IR-1000E, DAGE-MTI, Michigan, USA) and monitor. The patch pipette was filled with intracellular solution containing the following (in mM): 130 *K*-gluconate, 5 NaCl, 15 KCl, 0.4 EGTA, 10 HEPES, 4 Mg-ATP, and 0.2 Tris-GTP, pH 7.25–7.35, with an osmotic pressure of 290–300 mOsm/L. To visualize the recorded neurons, 0.2% biocytin (Sigma, USA) was added to the recording pipette solution. The pipette resistance, as measured in the bath, was typically 4 ± 0.5 MΩ. The voltage was held at -70 mV, and neurons were given at least 3 min to stabilize before data were collected. Spontaneous excitatory postsynaptic currents (sEPSCs), the paired-pulse ratio (PPR), and the number of action potentials (APs) were used to examine synaptic plasticity changes in the TMR-containing Vc-projecting neurons.

#### Spontaneous Discharge

Spontaneous excitatory postsynaptic currents were recorded at -70 mV in the presence of picrotoxin (100 mM, Sigma), a GABA_A_ receptor antagonist.

#### Paired-Pulse Ratio

To examine the PPR of EPSCs, paired pulses with an interval of 50 ms were delivered every 20 s. The PPR was defined as the ratio of the averaged amplitude of the second EPSC (EPSC2) to that of the first EPSC (EPSC1).

#### Spike Number

The membrane excitability of the recorded neurons was measured in current-clamp mode by determining the number of APs elicited by intracellular injection of 0-, 10-, 20-, 30-, 40-, 50-, and 60-pA depolarizing currents for 400 ms. The spike number was determined to estimate the influence of U0126 on the recorded neurons.

The access resistance was 15–30 MΩ initially and was monitored throughout the experiment. Data were filtered at 1 kHz and digitized at 10 kHz. The neurons were recorded using a Multiclamp 700B amplifier (Axon Instruments, Foster City, CA, USA). pCLAMP software (v. 10.02, Axon Instruments) was used to acquire and analyze the data. The signals were filtered at 2.6 kHz, digitized at 10 kHz (DigiData 1322A, Axon Instruments), and saved on a computer for oﬄine analysis.

### Statistical Analysis

Statistical data were analyzed using GraphPad Prism 5 software. The results are expressed as the mean ± standard error of the mean (SEM). Two-way analysis of variance (ANOVA) with Bonferroni’s multiple comparison tests or one-way ANOVA with Tukey’s multiple comparison *post hoc* tests were used for between-group comparisons (for example, the analysis of Western blot data with IoN-CCI surgery and drug administration as main effects). Student’s paired *t*-test was used to analyze the differences between two groups (for example, the difference in the 50% HWT between the IoN-CCI D14 + vehicle group and the IoN-CCI D14 + U0126 group). *P*-values < 0.05 were considered significant.

## Results

### IoN-CCI Produced Significant Mechanical Allodynia in Rats

**FIGURE 1:**
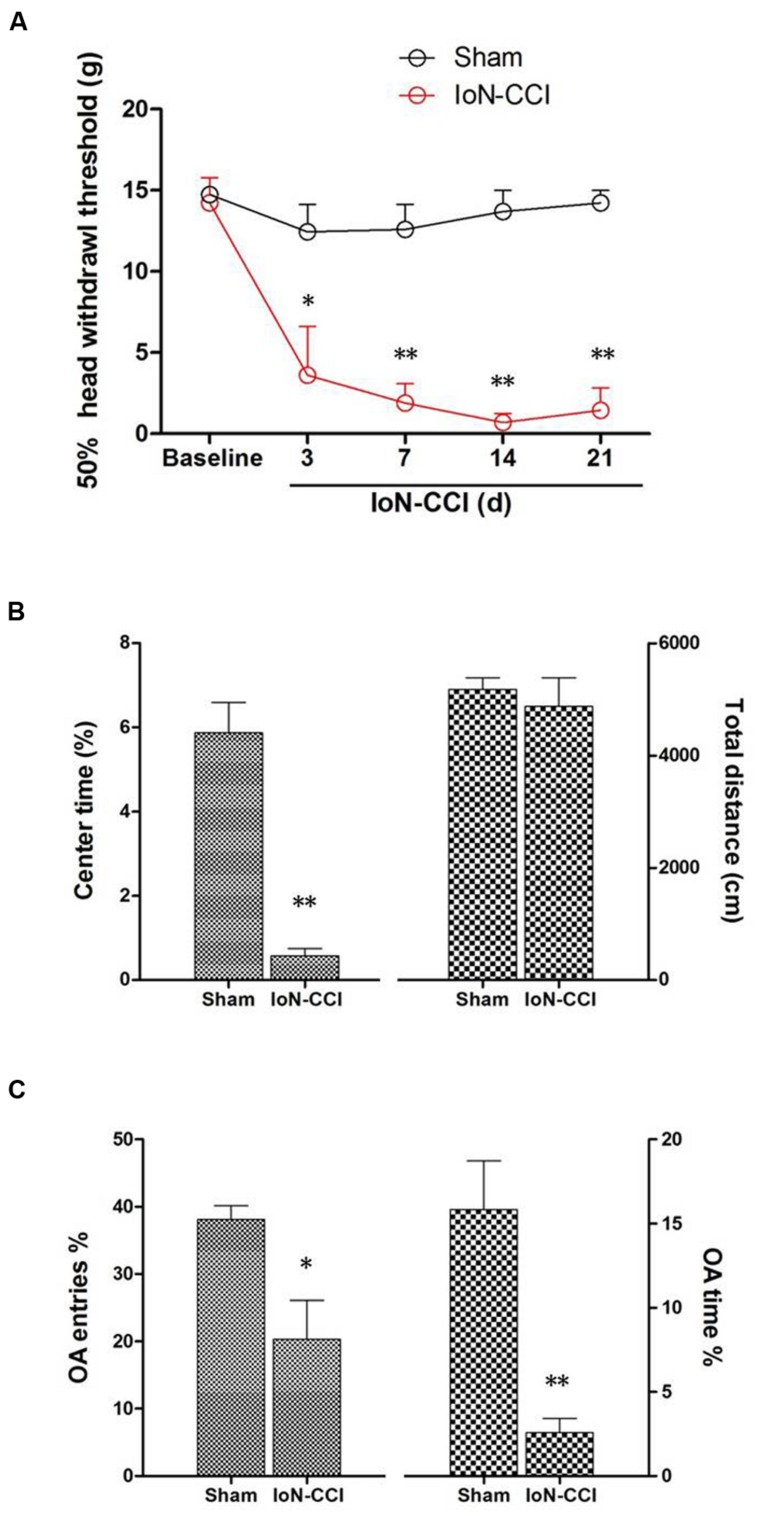
**Infraorbital nerve chronic constriction injury (IoN-CCI) produced significant nociceptive behaviors and negative emotions in rats. (A)** The 50% HWT assessed by von Frey filaments after IoN-CCI and sham surgery (*n* = 8/group); **(B,C)** IoN-CCI induced obvious negative emotions on day 14 after IoN-CCI surgery, as indicated by the open field (OF) **(B)** and EPM tests **(C)**. (^∗^*P* < 0.05, ^∗∗^*P* < 0.01 compared with the sham control at the same time point).

IoN-CCI rats showed significant mechanical allodynia from day 3 to day 21 after surgery compared with baseline and with the sham group (*P* < 0.05) (**Figure 1A**). However, animals in the sham group displayed no obvious changes in their responses to mechanical stimuli after surgery compared to baseline (*P* > 0.05) (**Figure 1A**).

### IoN-CCI Rats Showed Obvious Anxiety/Depression-Like Behaviors

Chronic pain, especially neuropathic pain, is always accompanied by increased negative emotions, including anxiety and/or depression (Zhuo, 2008). These negative emotions usually emerge in the late phase following nerve or tissue injury (Zhuo, 2008; Dai et al., 2011); therefore, we selected post-CCI-D14 to assess anxiety and/or depression by using the OF and EPM tests. No significant between-group difference in locomotion was revealed by assessing the total distance traveled during the 10-min recording time in the OF test (**Figure 1B**; *P* > 0.05). However, compared with the sham group, the IoN-CCI group showed significantly reduced center time% (**Figure 1B**; *P* < 0.01) in the OF test and OA Time% (**Figure 1C**; *P* < 0.01) and OA Entries% (**Figure 1C**; *P* < 0.05) in the EPM test; thus, the IoN-CCI rats exhibited obvious anxiety/depression-like behaviors.

### The IC Sent Direct Projections to the Contralateral Vc in Rats

**FIGURE 2:**
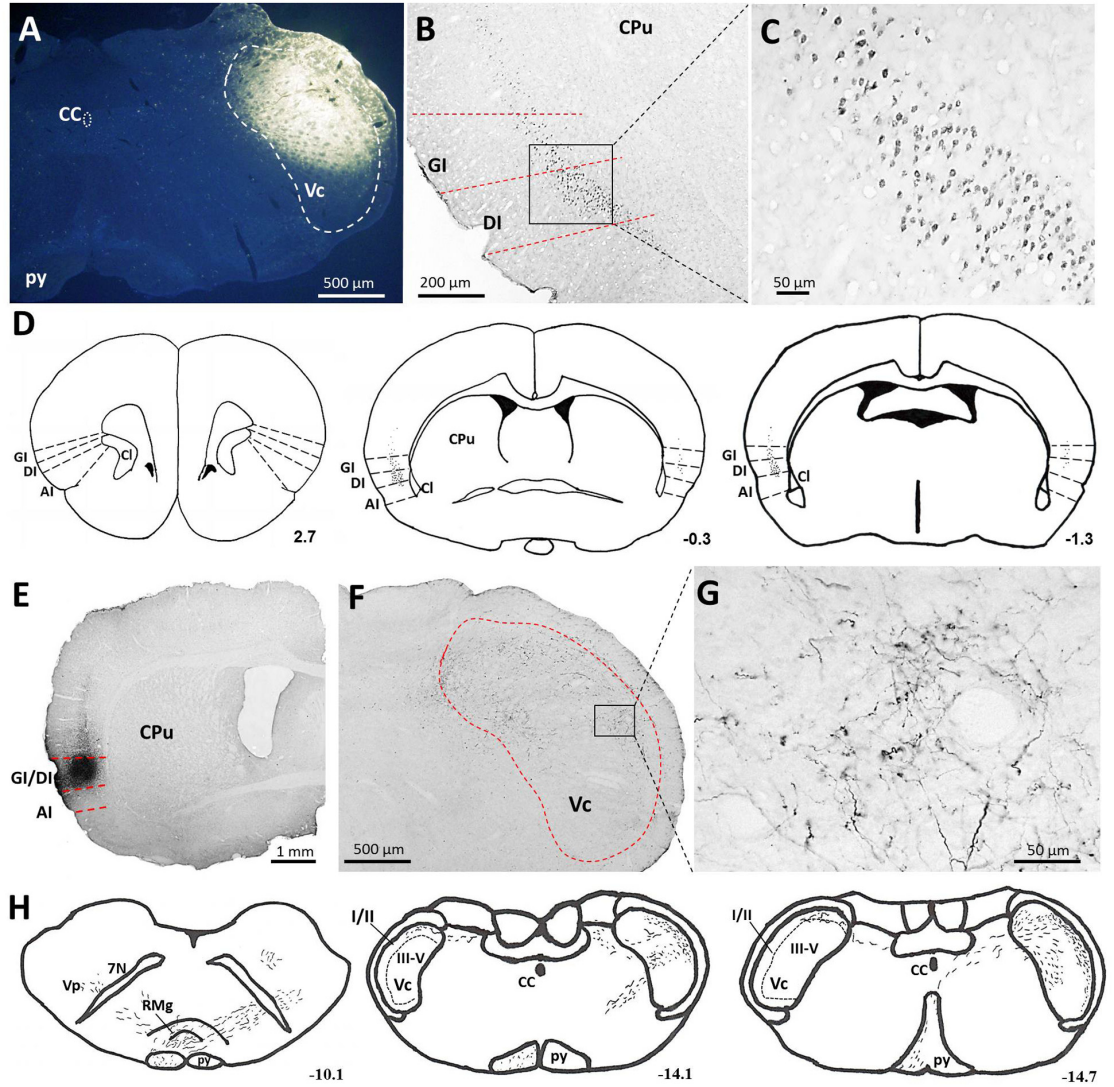
**Projections from the IC to the Vc. (A–D)** Photomicrographs showing FG injection sites in the Vc **(A)** of the brainstem and the resultant FG-labeled neurons in the IC **(B,C)**. **(B)** The retrogradely FG-labeled neurons were mainly distributed in the layer V of the GI and DI of the contralateral IC. **(C)** The rectangular area in **(B)** was enlarged and displayed in **(C)**. **(D)** Drawings of the distribution of retrogradely FG-labeled neurons in the IC after FG injection into the unilateral Vc. Retrogradely FG-labeled neurons, indicated by dots, were predominantly observed on the contralateral (left) side to the FG injection site. **(E–G)** Photomicrographs showing PHA-L injection sites **(E)** and the resultant PHA-L-labeled axonal fibers and terminals in the Vc of the brainstem **(F,G)**. **(F)** Anterogradely PHA-L-labeled axonal fibers and terminals originated from the GI/DI of the IC were observed on the contralateral side of laminae I-V of the Vc. **(G)** The rectangular area in **(F)** was enlarged and displayed in **(G)**. **(F)** Drawings of the distribution of anterogradely PHA-L-labeled axonal fibers and terminals in the Vc and other regions in the brainstem. Scale bar = 500 μm in **(A,F)**, 200 μm in **(B)**, 50 μm in **(C,G)**, and 1 mm in **(E)**. The numbers **(D,H)** correspond to the distance in millimeters (mm) posterior to bregma in the brain. CC, central canal; py, pyramidal tract; Vc, trigeminal caudal subnucleus; IC, insular cortex; GI, granular insular cortex; DI, dysgranular insular cortex; AI, agranular insular cortex; CPu, caudate putamen; Cl, claustrum; Vp, trigeminal principal nucleus; 7N, facial nerve; RMg, raphe magnus nucleus.

With the aim of identifying cortical regions projecting to the brainstem areas containing orofacial nociceptive neurons, the retrograde tracer FG was injected into the Vc (**Figure 2A**). FG injections into the superficial laminae (I/II) of the Vc produced dense retrograde labeling of a band of pyramidal cells located in layer V of all caudal but not rostral levels of the granular insular cortex (GI) and dysgranular insular cortex (DI). However, no FG-labeled neurons were observed in the agranular insular cortex (AI). These projections are bilateral, with obvious predominance in the contralateral side (**Figures 2B–D**).

To precisely determine the organization and distribution of IC afferents in the Vc, the anterograde tracer PHA-L was iontophoretically injected into the GI and DI regions based on our retrograde FG tract tracing results. PHA-L injections into the caudal GI/DI (**Figure 2E**) resulted in dense labeling in the contralateral Vc. Descending fibers from the IC were observed in the pyramidal tract; these fibers then crossed the rostral ventromedial medulla and terminated in the Vc (**Figures 2F–H**). A detailed view showed that the PHA-L-labeled fibers and terminals were scattered throughout the superficial (I/II) as well as the deeper laminae (V) of the Vc (**Figure 2G**).

### The p-ERK Signaling Pathway in the IC was Significantly Activated After IoN-CCI

**FIGURE 3:**
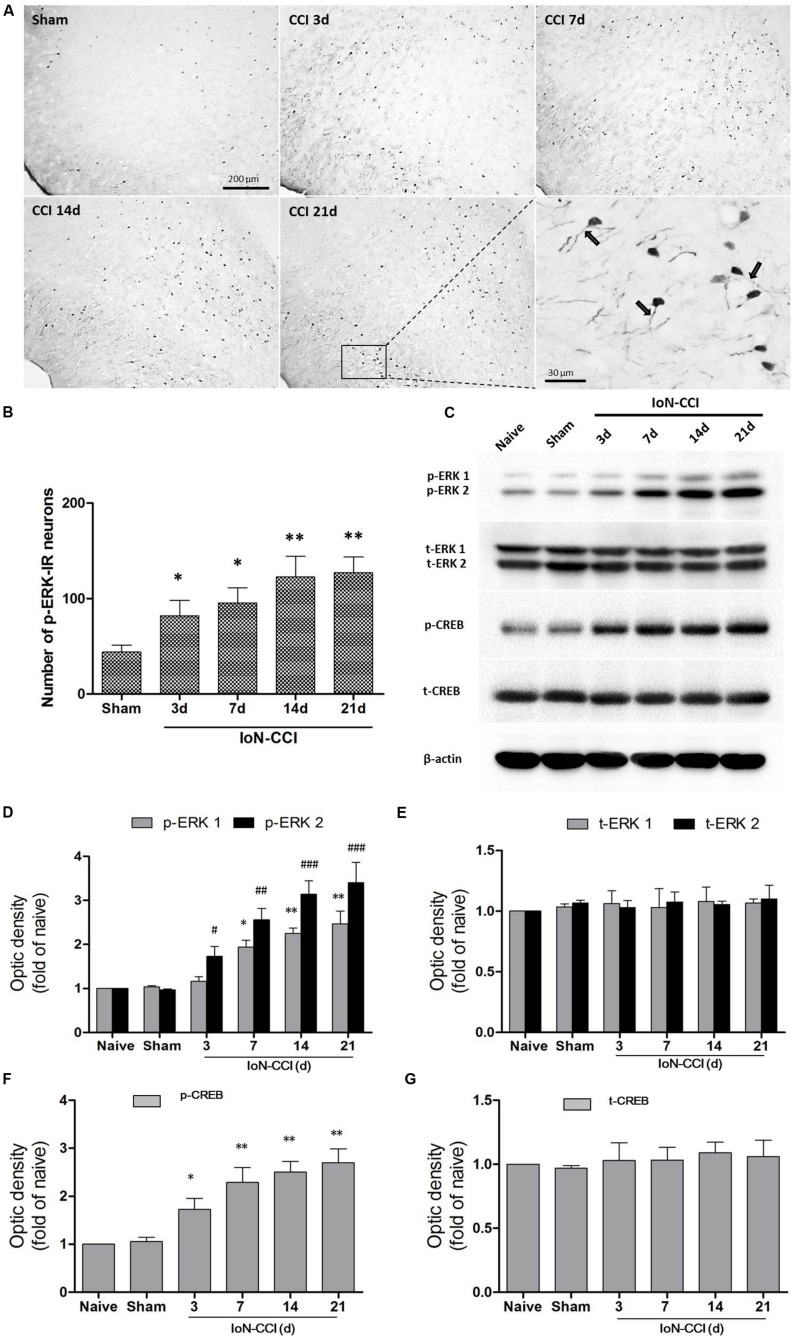
**The ERK pathway was remarkably activated in the IC after IoN-CCI. (A,B)** Phosphorylated ERK (p-ERK) expression was significantly increased from 3 through 21 days after IoN-CCI surgery (^∗^*P* < 0.05, ^∗∗^*P* < 0.01 compared with the sham control group). Scale bars = 200 μm or 30 μm, as indicated in the figures. Notably, the rectangular area in **(A)** was enlarged; the black arrows indicate the increased p-ERK expression within apical dendrites of the IC neurons 21 days after IoN-CCI. **(C–G)** Total and phosphorylated ERK **(C,D,E)** and CREB **(C,F,G)** expression was revealed by Western blotting. Three days after IoN-CCI, p-ERK (including p-ERK1 and p-ERK2) and p-CREB expression in the IC was significantly increased. Total ERK (t-ERK, including t-ERK1 and t-ERK2) and t-CREB expression was not changed after IoN-CCI (^∗^*P* < 0.05, ^∗∗^*P* < 0.01 compared with the p-ERK1 naïve control group; ^#^*P* < 0.05,^##^*P* < 0.01, ^###^*P* < 0.0001 compared with the p-ERK2 naïve control group in **(D)**; ^∗^*P* < 0.05, ^∗∗^*P* < 0.01 compared with the p-CREB naïve control group in F, *n* = 4)

According to previous studies, p-ERK expression in the spinal cord and supraspinal structures is highly correlated with neuroplasticity (Ji et al., 1999; Alvarez et al., 2009). Therefore, we explored p-ERK expression in the IC after the induction of neuropathic pain by IoN-CCI. The number of p-ERK-IR neurons was significantly increased in the IC from day 3 through day 21 in the IoN-CCI group compared with that in the sham group (**Figures 3A,B**), indicating that ERK activation in the IC may contribute to IoN-CCI-induced neuropathic pain. Moreover, consistent with previous work (Wei et al., 2008), increased p-ERK expression was also observed in the apical dendrites of neurons in the IC, especially in the late phase of neuropathic pain (**Figure 3A**).

cAMP response element binding protein (CREB), a main downstream plasticity-related protein activated by ERK, is involved in several intracellular processes, including neuronal plasticity and long-term memory (Zhuo, 2008). Therefore, we used Western blot analysis to further evaluate ERK signaling pathway members, including ERK and CREB, in the contralateral IC after IoN-CCI surgery. Compared with naive control rats, the expression of both phosphorylated ERK and phosphorylated CREB (p-ERK and p-CREB) was not significantly altered in the sham rats (**Figures 3C,D,F**). As indicated in **Figures 3C,D**, p-ERK and p-CREB were significantly elevated in the IC 3 days after IoN-CCI compared with their levels in the control group, and phosphorylation was maintained for at least 21 days (**Figure 3**; p-ERK1: IoN-CCI D7: 1.94 ± 0.21; IoN-CCI D14: 2.26 ± 0.19; IoN-CCI D21: 2.49 ± 0.48-fold of naive control, ^∗^*P* < 0.05, ^∗∗^*P* < 0.01; p-ERK2: IoN-CCI D3: 1.72 ± 0.35; IoN-CCI D7: 2.63 ± 0.42; IoN-CCI D14: 3.14 ± 0.48; IoN-CCI D21: 3.47 ± 0.52-fold of naive control,^#^*P* < 0.05, ^##^*P* < 0.01, ^###^*P* < 0.0001; p-CREB: IoN-CCI D3: 1.75 ± 0.23; IoN-CCI D7: 2.16 ± 0.37; IoN-CCI D14: 2.51 ± 0.20; IoN-CCI D21: 2.81 ± 0.32-fold of naïve control, ^∗^*P* < 0.05,^∗∗^*P* < 0.01, *n* = 4). The change in p-CREB was similar to that in p-ERK, indicating that the ERK signaling pathway in the IC was activated by IoN-CCI 3 days after surgery. Total ERK and CREB levels were not changed in any of the groups in the present study (**Figures 3C,E,G**). In summary, the ERK signaling pathway, including ERK and CREB, was activated in the IC, perhaps indicating the occurrence of new protein synthesis and resulting in changes in neuroplasticity.

### Infusion of U0126 Rapidly Decreased the Upregulation of p-ERK in the IC and the Expression of Fos in the Vc in IoN-CCI Rats

**FIGURE 4:**
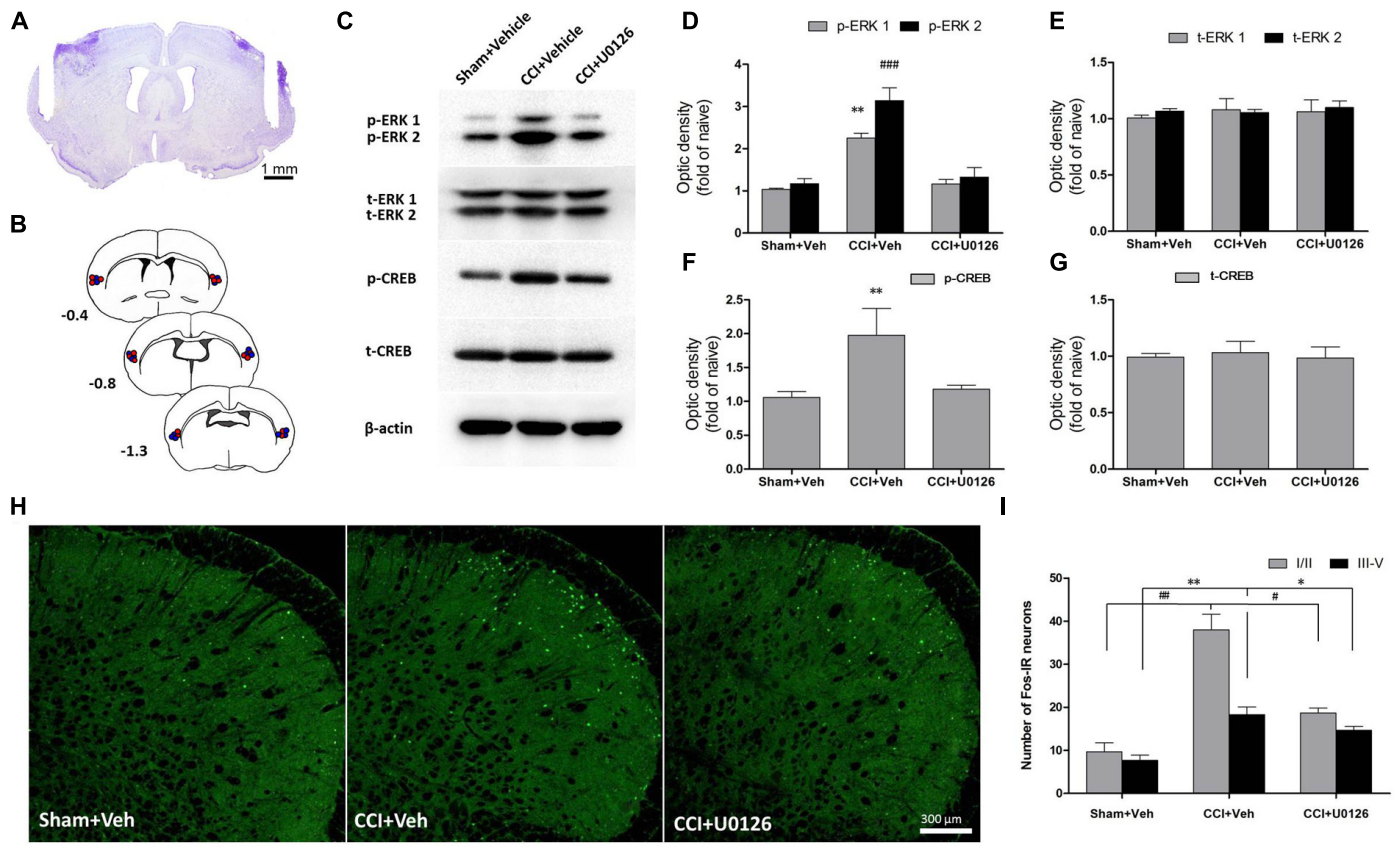
**Intra-IC U0126 microinjection reduced ERK pathway activity in the IC and decreased Fos expression in the Vc. (A)** Representative Nissl-stained section showing injection sites within the IC. Scale bar = 1 mm. **(B)** Camera lucida drawings showing cannula tip placements in rats injected with U0126 (blue circles, *n* = 16) or vehicle (red circles, *n* = 16) in the IC. The numbers correspond to the distance in millimeters (mm) posterior to bregma in the brain. **(C–G)** Intra-IC U0126 infusion effectively inhibited ERK activation in IoN-CCI rats. In the absence of U0126, IoN-CCI produced a significant increase in the levels of phosphorylated ERK (p-ERK1/2) and CREB (^∗∗^*P* < 0.01; ^###^*P* < 0.001 compared to the sham + vehicle group, *n* = 4). U0126 treatment remarkably decreased the upregulation of p-ERK (p-ERK1/2) and p-CREB expression in the IC of IoN-CCI rats. **(H,I)** Intra-IC U0126 administration partially reversed the elevated Fos expression in both laminae I/II and III-V of the Vc after IoN-CCI (^∗∗^*P* < 0.01 ^##^*P* < 0.01 CCI + vehicle compared with the sham + vehicle group; ^∗^*P* < 0.05 ^#^*P* < 0.05 CCI + vehicle compared with the CCI + U0126 group; *P* < 0.05 CCI + U0126 compared with the sham + vehicle group, *n* = 4).

Because ERK is activated after IoN-CCI in the IC, we microinjected the inhibitor of ERK activation U0126 into the bilateral IC (**Figure 4A**) to test whether p-ERK expression could be reduced. One hour after microinjection, the IC regions were collected and assessed by Western blotting. IoN-CCI significantly increased the expression of both p-ERK and its downstream molecule, p-CREB (**Figures 4C–G**, p-ERK1: IoN-CCI D14 + vehicle: 2.23 ± 0.18-fold of naïve control, *^∗∗^P* < 0.01; p-ERK2: IoN-CCI D14 + vehicle: 3.08 ± 0.34-fold of naïve control, *^###^P* < 0.001; p-CREB: IoN-CCI D14 + vehicle: 1.94 ± 0.47-fold of naive control, *n* = 4, ^∗∗^*P* < 0.01), which is consistent with previous Western blot data ((**Figure 3**). Moreover, U0126 significantly reversed the upregulation of p-ERK and p-CREB after 1 h of drug infusion (p-ERK1: IoN-CCI D14 + U0126: 1.16 ± 0.17-fold of naive control *vs.* IoN-CCI D14 + vehicle: 2.23 ± 0.18-fold of naive control, *P* < 0.05; p-ERK2: IoN-CCI D14 + U0126: 1.21 ± 0.29-fold of naive control *vs.* IoN-CCI D14 + vehicle: 3.08 ± 0.34-fold of naive control, *P* < 0.05; p-CREB: IoN-CCI D14d + U0126: 1.17 ± 0.13-fold of naive control *vs.* IoN-CCI D14 + vehicle 1.94 ± 0.47-fold of naive control, *n* = 4, *P* < 0.05), indicating that U0126 could effectively inhibit ERK activation in the IC after IoN-CCI *in vivo*.

Based on our observation that the IC could send direct projections to the contralateral Vc, we thus investigated the nuclear expression of Fos, a neuronal activation marker, in the Vc after the bilateral intra-IC injection of U0126 in rats with IoN-CCI-induced neuropathic pain. Neuronal activation was significantly increased, as indicated by the number of nuclei expressing Fos on day 14 after IoN-CCI surgery (**Figures 4H,I**; IoN-CCI D14 + vehicle *vs*. sham + vehicle, *^∗∗^P* < 0.01, *^##^P* < 0.01). Moreover, these Fos-IR neurons were located within both laminae I/II and III-V in vehicle-treated IoN-CCI rats. However, the number of Fos-IR neurons was significantly reduced following intra-IC U0126 administration compared with that following vehicle treatment (**Figures 4H,I**; IoN-CCI D14 + U0126 vs. IoN-CCI D14 + vehicle, *^∗^P* < 0.05, *^#^P* < 0.05), which indicated that intra-IC U0126 administration could decrease the elevated neuronal activation in the Vc after IoN-CCI. Nonetheless, the number of Fos-IR neurons after U0126 microinjection remained higher than the sham + vehicle group (**Figures 4H**, I; IoN-CCI D14 + U0126 *vs.* sham + vehicle, *P* < 0.05), suggesting that the reduction of Fos expression by intra-IC U0126 occurs partially but not completely in the Vc.

### U0126 Decreased Both the Frequency and Amplitude of sEPSCs and Reduced the PPR and Excitability of Vc-Projecting Neurons in IoN-CCI Rat Slices *In Vitro*

**FIGURE 5:**
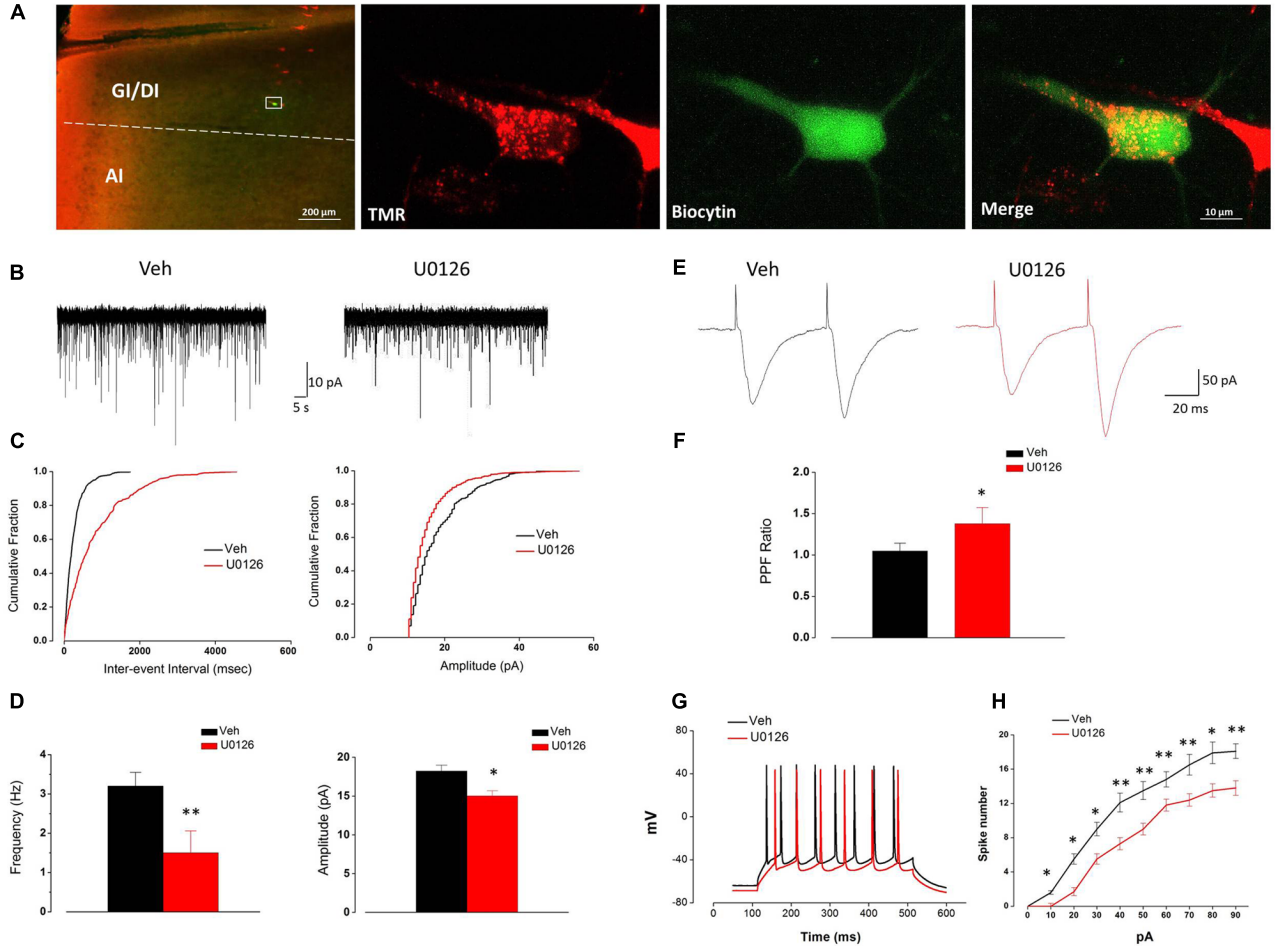
**The activity of Vc-projecting neurons in the IC of IoN-CCI rats was effectively impaired by U0126 (5 mM) perfusion. (A)** A representative retrogradely TMR-labeled neuron (red) in the IC was recorded and injected with biocytin (labeled with Alexa 488 (green). The white rectangle area in the first panel is enlarged in the following three panels. (GI, granular insular cortex; DI, dysgranular insular cortex; AI, agranular insular cortex). Scale bar = 200 μm (left) and 10 μm (right). **(B–D)** Superimposed samples and cumulative fraction results showing that U0126 significantly inhibited both the frequency and amplitude of the sEPSCs of Vc-projecting neurons in the IC of IoN-CCI rats. **(B,C)** Bath application of U0126 (5 mM, 1 h) inhibited the frequency and amplitude of sEPSCs in rats with IoN-CCI. **(D)** Summary of the effects of U0126 on the sEPSCs of Vc-projecting neurons in IoN-CCI rats (U0126 *vs*. vehicle, ^∗^*P* < 0.05, ^∗∗^*P* < 0.01; *n* = 15). **(E,F)** Effects of U0126 on the PPR of paired EPSCs in IoN-CCI rats. Paired EPSCs were evoked by the delivery of paired pulses with an inter-pulse interval of 50 ms, and the PPR was measured as the ratio of the averaged amplitude of the second EPSC to that of the first EPSC. **(E)** Traces of paired EPSCs in representative neurons that were perfused with vehicle or U0126. **(F)** The averaged PPR recorded in the presence of vehicle or U0126 (U0126 *vs*. vehicle, ^∗^*P* < 0.05; *n* = 10). **(G,H)** Sample traces and average results showing that the number of action potentials (APs) in a train induced by the injection of step currents (400 ms, 0–90 pA) was significantly reduced by U0126 in IoN-CCI rats (*n* = 15, *P* < 0.05, two-way repeated measures ANOVA). The Holm-Sidak *post hoc* test indicated that U0126 decreased the spike number when currents of 10 pA (*t* = 2.50, ^∗^*P* < 0.05), 20 pA (*t* = 2.62, ^∗^*P* < 0.05), 30 pA (*t* = 2.34, ^∗^*P* < 0.05), 40 pA (*t* = 3.34, ^∗∗^*P* < 0.01), 50 pA (*t* = 2.50, ^∗∗^*P* < 0.01), 60 pA (*t* = 3.34, ^∗∗^*P* < 0.01), 70 pA (*t* = 3.62, ^∗∗^*P* < 0.01), 80 pA (*t* = 2.57, ^∗^*P* < 0.05), and 90 pA (*t* = 5.07, ^∗∗^*P* < 0.01) were applied.

Consistent with our FG retrograde labeling results, TMR-labeled Vc-projecting neurons were mainly found in layer V of the GI/DI portions of the IC (data not shown). To further investigate the effect of U0126 on the excitability of Vc-projecting neurons in the IC, we made whole-cell patch-clamp recordings from the TMR-labeled Vc-projecting neurons (**Figure 5A**). The effects of U0126 on sEPSCs were studied in 15 Vc-projecting neurons. Perfusion of U0126 (5 mM) for 1 h considerably reduced the frequency and amplitude of sEPSCs (**Figures 5B–D**). Overall sEPSC frequency was significantly reduced from 3.28 ± 0.35 Hz in the vehicle control to 1.51 ± 0.54 Hz in the presence of U0126 (*P* < 0.01, *n* = 15; **Figure 5D**). sEPSC amplitude was also reduced from 18.26 ± 0.72 pA in the vehicle control to 15.05 ± 0.64 pA in the presence of U0126 (*P* < 0.05, *n* = 15; **Figure 5D**). These results suggested that U0126 could decrease sEPSCs in IC slices of IoN-CCI rats via both pre- and postsynaptic mechanisms.

Due to the notable decrease in the frequency of sEPSCs, we subsequently compared the EPSC PPR to measure presynaptic transmitter release changes in response to U0126 and vehicle. One hour after U0126 (5 mM) perfusion, the PPR was significantly increased (*n* = 10, *P* < 0.05) (**Figures 5E,F**), suggesting that presynaptic release of glutamate onto the Vc-projecting neurons was likely decreased by U0126 administration in the IoN-CCI rat slices.

We next tested the effects of U0126 on the APs of the Vc-projecting neurons. The spike numbers of the recorded neurons in the IC slices of IoN-CCI rats were significantly reduced in the presence of U0126 (5 mM) compared to vehicle (*n* = 15, ^∗^*P* < 0.05, ^∗∗^*P* < 0.01) (**Figures 5G,H**). These results indicate that U0126 inhibited the excitability of the Vc-projecting neurons in the IC under neuropathic pain conditions.

Taken together, these results suggest that U0126 inhibited the Vc-projecting neurons in the IC via both pre- and postsynaptic mechanisms after IoN-CCI.

### The Microinjection of U0126 into the Bilateral IC not only Alleviated Nociceptive Behaviors but also Decreased Negative Emotions in IoN-CCI Rats

**FIGURE 6:**
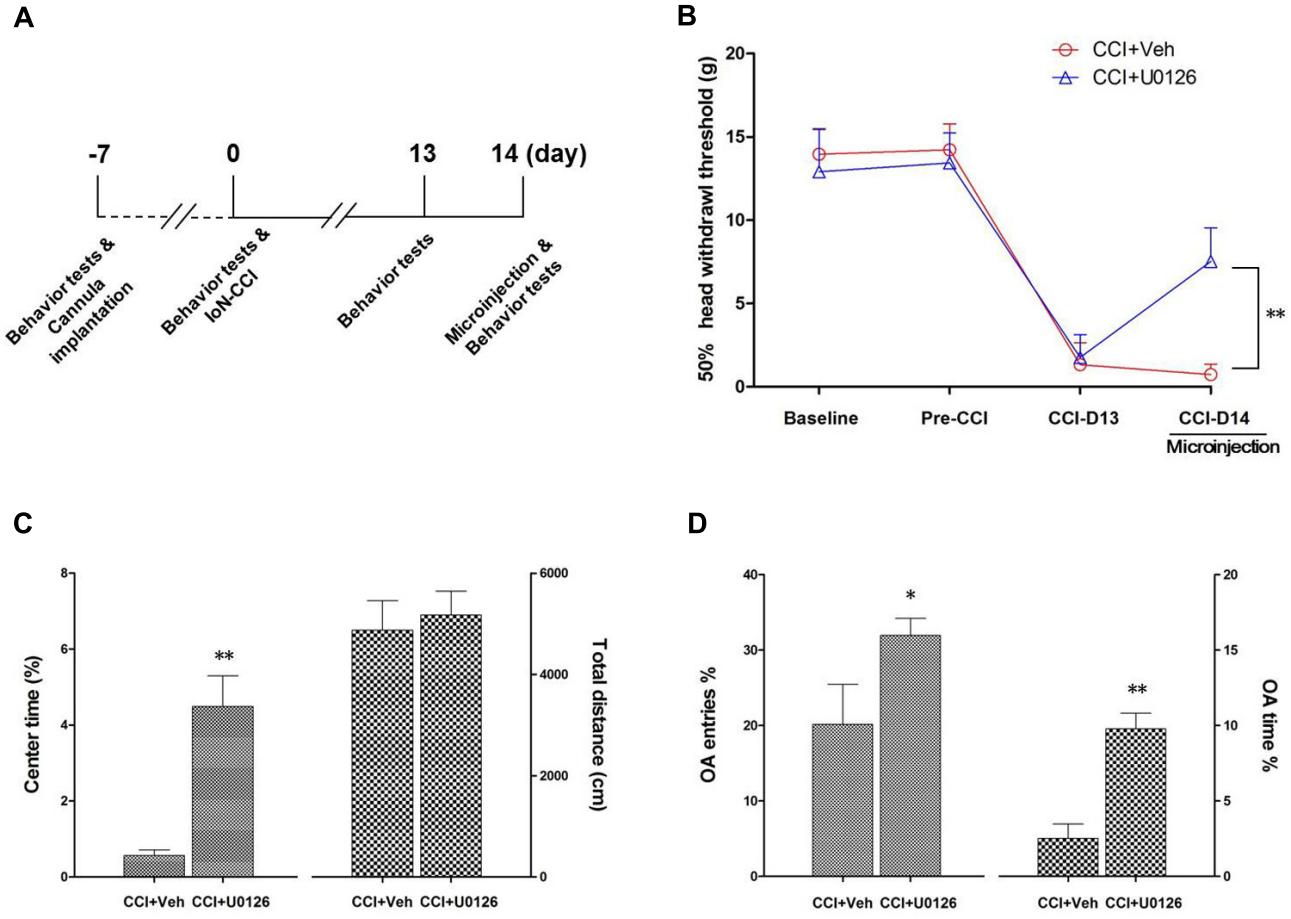
**Intra-IC U0126 microinjection improved nociceptive behaviors as well as pain-related negative emotions. (A)** Experimental schedule. The behavioral tests, cannula implantation, microinjection, and IoN-CCI surgery were conducted as indicated in the schedule. **(B)** The 50% HWT were significantly improved by bilateral intra-IC U0126 microinjection following IoN-CCI (^∗∗^*P* < 0.01, CCI + U0126 compared with CCI + vehicle group, *n* = 4 rats/group). **(C,D)** Pain-related negative emotions such as anxiety/depression-like behaviors, as revealed by the OF **(C)** and EPM **(D)** tests, were significantly improved after intra-IC U0126 microinjection on day 14 after IoN-CCI (^∗^*P* < 0.05; ^∗∗^*P* < 0.01, CCI + U0126 compared with CCI + vehicle group, *n* = 4 rats/group).

To determine whether U0126 could alleviate IoN-CCI-induced nociceptive behaviors as well as negative emotions in rats, U0126 was microinjected via a cannula implanted into the bilateral IC (**Figures 4A,B**) on day 14 after IoN-CCI, and behavioral testing was conducted 1 h later. On day 13 after IoN-CCI, the rats demonstrated typical increased nociceptive responses to non-noxious mechanical stimulation (**Figure 6B**). Compared with the vehicle control group, the mechanical allodynia revealed by 50% HWT was significantly reduced after U0126 microinjection into the IC on day 14 after IoN-CCI (**Figure 6B**, *^∗∗^P* < 0.01). Thus, evoked nociceptive behaviors were significantly reduced following the intra-IC microinjection of U0126 in IoN-CCI rats.

To investigate the effect of intra-IC U0126 microinjection on negative emotions in IoN-CCI rats, we subsequently conducted the OF and EPM tests. Because rats showed obvious anxiety/depression-like behaviors on day 14 after IoN-CCI (**Figure 1**), we performed behavioral tests on IoN-CCI D14. No significant difference was found between the IoN-CCI D14 + U0126 and IoN-CCI D14 + vehicle groups in locomotion based on the total distance traveled during the 10-min recording time in the OF test (**Figure 6C**; *P* > 0.05). However, compared with vehicle, U0126 significantly increased the center time% (**Figure 6C**; *P* < 0.01) in the OF test as well as the OA Entries% (**Figure 6D**; *P* < 0.05) and OA Time% (**Figure 6D**; *P* < 0.01) in the EPM test, indicating that intra-IC U0126 microinjection improved anxiety/depression-like negative emotions.

## Discussion

Our present study demonstrates for the first time that direct corticotrigeminal projections from the IC to Vc are involved in IoN-CCI-induced orofacial neuropathic pain. Specifically, this descending projection may regulate Vc sensory neurons, thus exasperating neuropathic pain. Moreover, perfusion of IC slices of the IoN-CCI rats with the ERK inhibitor U0126 significantly reduced the activity of Vc-projecting neurons. Finally, intra-IC microinjection of U0126 effectively alleviated not only nociceptive behaviors but also accompanied negative emotions in IoN-CCI rats.

### IoN-CCI Induced Neuropathic Pain and Related Negative Emotions

In this study, IoN-CCI led to significant changes in responses evoked by mechanical stimulation of the face. Additionally, consistent with previously reports that tissue or nerve injury could induce anxiety/depression-like behaviors in rodents (Dai et al., 2011; Lyons et al., 2015), our results also demonstrated that IoN-CCI could produce pain-related negative emotional behaviors such as anxiety/depression, as revealed by the OF and EPM tests.

### ERK Pathway Activation in the IC After IoN-CCI

Extracellular signal-regulated kinase, one important MAPK superfamily member, has been extensively studied in tumors (Deschenes-Simard et al., 2014), cardiovascular diseases (Lorenz et al., 2009), and regeneration (Sellers et al., 2015). Recent emerging evidence indicates that ERK plays a significant role in pain processing, and ERK is important in the regulation of nociception at both the spinal cord and supraspinal levels. In the spinal cord, ERK activation in dorsal horn neurons is essential for the development of central sensitization, which is responsible for the generation and maintenance of persistent pain (Ji et al., 2009). In the amygdala, studies have confirmed an important role of ERK in the CeA for pain-related synaptic plasticity and behavior (Fu et al., 2008). Moreover, ERK activation in the ACC is involved in formalin-induced inflammation-related pain (Wei et al., 2008), and IC ERK phosphorylation contributes to the mechanisms underlying abnormal pain perception under nerve injury induced neuropathic pain conditions (Alvarez et al., 2009).

Once activated, p-ERK can be translocated into the nucleus, thus activating several transcriptional factors such as CREB, which is required for the transcription of several neuronal genes and for long-term synaptic plasticity (Ji and Rupp, 1997). The ERK-CREB pathway likely increases the activity as well as the membrane insertion of excitatory glutamate receptors (AMPA and NMDA) (Kawasaki et al., 2004) and suppresses inhibitory potassium channel (Kv4.2) activity (Sutton et al., 2004), thus producing the LTP of neurons in many pain perception regions under chronic pain conditions.

In the current study, we demonstrated that the ERK pathway was significantly activated in the IC from 7 to 21 days after IoN-CCI surgery. Intra-IC microinjection of U0126, a potent and selective MEK/ERK inhibitor (Favata et al., 1998) that does not affect other major signal transduction pathways (Roberson et al., 1999), effectively alleviated established mechanical allodynia. Our data here support previous reports showing that the IC is a critical cortical region in pain regulation (Coffeen et al., 2011; Qiu et al., 2013); additionally, the ERK pathway is indeed involved in neuropathic pain in cortical regions (Cao et al., 2009). More importantly, we demonstrated why the deactivation of IC neurons via the inhibition of ERK phosphorylation could produce an obvious amelioration of orofacial neuropathic pain induced by IoN-CCI.

### A Direct Corticotrigeminal Descending Modulation Pathway from the IC to the Vc

According to previous studies, the IC could send projecting fibers to the trigeminal complex, including the Vc (Noseda et al., 2010; Sato et al., 2013). However, in contrast, several studies reported the opposite negative results (Jasmin et al., 2004; Benison et al., 2011). Consequently, the connections between the IC and the Vc remain quite controversial. In the present study, by using anterograde and retrograde tract tracing methods, we clearly demonstrated that the deep layer (V) of the GI and DI sent direct descending projections to the contralateral Vc, especially to the superficial laminae (I/II) in the brainstem. Our data support earlier studies showing the existence of direct projections from the GI/DI to the contralateral Vc (Noseda et al., 2010; Sato et al., 2013). As orofacial nociceptive primary afferents terminate in laminae I/II of the Vc (Dubner and Bennett, 1983), this top–down corticotrigeminal projection from the GI/DI to laminae I/II of the Vc are likely involved in orofacial nociceptive processing. Notably, according to the current results, the Vc received no projections from the AI, which received much attention as a cortical center of nociception in previous reports (Jasmin et al., 2004; Alvarez et al., 2009; Coffeen et al., 2011); thus, the AI may not be involved in orofacial pain processing, at least in this top–down direct modulation pathway.

### Decreased Activity of Vc-Projecting Neurons in the IC through the Deactivation of ERK

We explored the effects of U0126 on the Vc-projecting neurons of IoN-CCI rats by whole-cell patch-clamp recording *in vitro* and found that U0126 could inhibit the activity of these neurons through both pre- and postsynaptic mechanisms. Specifically, U0126 decreased not only the amplitude but also the frequency of sEPSCs. Furthermore, the PPR, which depends on the calcium-dependent presynaptic release machinery, was significantly increased after U0126 perfusion. These data indicate that the inhibition of ERK leads to both pre- and postsynaptic plasticity changes in Vc-projecting neurons. Similar results were reported in previous studies in the amygdala (Fu et al., 2008) and somatosensory cortex (Arendt et al., 2004). Additionally, the number of APs was also significantly reduced in the presence of U0126, suggesting a decrease in the excitability of IC neurons in the IoN-CCI rats. According to our morphological data, p-ERK is expressed throughout the IC, from layer II to V, in IoN-CCI rats. Moreover, neurons in cortical layer II/III are thought to receive sensory inputs from other brain regions and to send projections to layer V neurons, which are the main output region and project to subcortical areas. Consequently, U0126 perfusion could affect p-ERK-containing neurons in both layers II/III and V in the IC slices. As a result, ERK deactivation-induced pre-and postsynaptic plasticity alterations were observed in the Vc-projecting neurons that were recorded in the present study.

### Reduced Nociception and Negative Emotions via ERK Deactivation in the IC

The deactivation of ERK in the IC through intra-IC U0126 administration resulted in decreased Fos expression in the Vc in IoN-CCI-induced neuropathic pain, indicating the inhibition of activated Vc sensory neurons. Together with the tract tracing data, these data indicate that that projection neurons of the IC may send excitatory projecting fibers (glutamatergic) and terminate in the Vc, especially in the superficial laminae (I/II), to regulate local orofacial nociceptive transmission. IoN-CCI surgery ultimately exerts a facilitatory effect on this pathway, which in turn further exaggerates nociceptive transmission and leads to chronic orofacial pain. In the present study, intra-IC U0126 microinjection could inhibit ERK pathway activation and limit the subsequent synaptic plasticity that occurred under neuropathic pain conditions. As a result, the top–down corticotrigeminal descending excitatory projections from the IC to the Vc were prevented, and pain facilitation was diminished.

Based on the findings of the current study, not only nociceptive behaviors but also anxiety/depression-like negative emotions were improved by intra-IC ERK inhibition in IoN-CCI rats. Indeed, similar to the ACC, the IC is a complicated integration site for sensations and affections (Zhuo, 2008). The IC receives afferent projections from thalamic nuclei and forms reciprocal connections with the amygdala, mPFC, and limbic system (Jasmin et al., 2004), all of which are related to emotions and affections in animals and human. Therefore, targeting the IC is promising for the achievement of multiple purposes, including the regulation of pain sensation as well as pain-related affections. In a previous study, the intra-ACC administration of an ERK inhibitor attenuated pain-related anxiety in a postoperative pain model (Dai et al., 2011). Furthermore, our group has previously demonstrated that inhibiting ERK phosphorylation by the microinjection of U0126 into the mPFC attenuated stress-induced anxiety/depression-like behaviors (Qi et al., 2014). These results are in line with those of the present study, indicating that anxiety/depression-like negative emotions are improved following intra-IC U0126 microinjections in IoN-CCI rats.

Our study also has some limitations. Conditioned place preference test should be further utilized to validate the effects of U0126 on the spontaneous/ongoing pain of IoN-CCI rats. Due to the lack of a specific ERK activator, reverse experiments involving ERK activation in the IC, which should exaggerate orofacial neuropathic pain, are difficult to conduct. Moreover, optogenetic methods as well as transgenic animals should be further employed to confirm the role of ERK in the corticotrigeminal descending modulation pathway in both neuropathic and inflammatory pain.

## Conclusion

In summary, our present study offers powerful evidence that a novel direct corticotrigeminal descending modulation pathway from the IC to the Vc regulates IoN-CCI-induced orofacial neuropathic pain. Because ERK deactivation in the IC weakens descending pain facilitation, specifically targeting ERK activation in the IC represents a promising avenue for the management of neuropathic pain as well as pain-related negative emotions.

## Author Contributions

JW, Z-HL, and BF contribued equally to this manuscript as first authors. JC, W-DZ, and Y-QL designed the experiments. JW, Z-HL, and TZ performed the animal model and cannula implantation surgury. Z-HL, BF, and HZ conducted the behavioral tests. JW and Z-HL performed the Western blot assays. JW and Z-HL did the tract tracing experiments. JW, Z-HL, and TZ carried out the immunostaining experiments. BF performed the electrophysiology study. HL, TC, and W-DZ revised the manuscript. JW, JC, W-DZ, and Y-QL wrote the manuscript. All authors discussed the manuscript. All authors read and approved the final manuscript.

## Conflict of Interest Statement

The authors declare that the research was conducted in the absence of any commercial or financial relationships that could be construed as a potential conflict of interest.
